# Annotations

**Published:** 1904-05-28

**Authors:** 


### ANNOTATIONS.

### Lunacy and Pauper Statistics.

In a paper lately submitted by Mr. R. J. Thompson to the Royal Statistical Society there are presented several series of figures which indicate in a very striking fashion certain tendencies of modern social development. After reference to the rapid growth of expenditure and indebtedness of local authorities which has marked the course of recent years, Mr. Thompson quotes the charges for Poor-law relief and lunatic asylums and shows that these have risen from £7,644,000 in 1869–70 to £12,891,000—an increase of nearly 60 per cent. During the same period the expenses for lunatics confined in asylums have risen from £723,000 to £2,045,000. This coincides with a considerable increase in the number of lunatics, but before concluding that such a result means an actual increase of lunacy it is necessary to remember that many persons are now certified who twenty years ago would have been kept at home by their relatives. Just as the poorer classes have learned to welcome the public hospitals in cases of infectious disease so they have, at least to a large extent, lost their prejudice against asylums for the reception of those who are of unsound mind. Further, the public conscience has demanded a much improved standard of comfort for those confined in public institutions, and this is well shown in the in-maintenance and out-relief of paupers. No increase in the number of indoor paupers has occurred in the last 30 years, but the cost per head has risen from £9 12s. to £13 6s. A satisfactory point to note is that in the absolute numbers of those in receipt of out-door relief there has been a decrease from 848,000 in 1869–70 to 501,000 in 1901–2. The misleading tendency of figures is a familiar popular remark, but those interested in social science will recognise that in the above statistics, as well as in many others related in the paper from which we quote, there exists a sure index of real movements which greatly concern the national welfare.

### The Care of the Russian Wounded.

Three weeks ago the President of the Red Cross Society for Japan, in forwarding The Hospital an account of the provision for the nursing of the Japanese troops, gave some interesting details of the gratitude manifested by several wounded Russian sailors who, in their hour of need, were cared for by "their friends, the enemy." There is further testimony this week, from St. Petersburg, of the kindness and skill shown by the Japanese in their treatment of the Russian wounded. Two injured men of the 11th Russian Regiment, which suffered so severely at Kiu-lien-cheng, managed to escape through the Japanese line, and have reached Mukden. They report, in enthusiastic terms, as to the management of the Japanese field-hospitals, and the attention given to between four and five hundred Russians in the Japanese camp. It looks as if the story of the British and Boers were being repeated, for just as the Boer soldiers declared that the treatment of their wounded by the British contributed to the formation of good feeling, so the Russian troops affirm that the relations between themselves and the Japanese on neutral ground are excellent. It is satisfactory to learn that Surgeon Wilson, who was invited by the Captain of the United States steamship *Vicksburg* to assist in the care of the wounded on board the Russian warship at Chemulpo, reports that the accommodation provided for the men was good, and that the United States warship rendered all the assistance to the Russian wounded that was possible. The latest movement of the Russians themselves to provide further aid for their wounded, which is badly needed, is the utilisation of dogs. The German Emperor has presented three Scotch sheep-dogs, who have been trained in ambulance work, to the Russian Dog Breeders' Association, and after they have been tested they will be sent to the Far East. It is also intended by the Association to teach a number of animals to be of assistance, either by remaining at the side of a wounded man when they have found him and attracting the attention of the ambulance by barking, or by going silently in search of the ambulance corps and conducting them to the spot where the wounded man is lying. Each animal is to carry a wallet strapped on its back, containing bandages, restoratives, and water. That dogs may render valuable service in war was proved by the Germans during the Boxer outbreak in China, but the Russian authorities will not, it may be hoped, be content until they have brought their medical, surgical, and nursing arrangements generally more up to the Japanese standard. At present, the wounded Russian who falls into the hands of the foe is much more likely to fare well than he who has to rely upon the ministrations of Muscovites.

### Degrees in Veterinary Science.

The medical profession will certainly not look with any jealous eye on the efforts at present being made by the authorities responsible for the various veterinary colleges of the United Kingdom to obtain for their students the dignity and advantages of a University degree. It has become of recent years increasingly evident that a survey of pathology from the comparative standpoint is likely to yield considerable assistance in the attempted solution of some of the most obscure disease problems, and hence those concerned more especially with human pathology will be disposed to lend their support to a proposal which may direct on to more scientific lines the study of the diseases of the lower animals. There is a broad stretch of ground common to the two professions and it is more than sufficient to sustain a mutual sympathy. Both in London and Dublin it has been intimated that the University authorities are prepared to grant degrees to veterinary students. In Edinburgh the Royal Veterinary College has so far failed to produce a parallel result, and from a recent speech by Principal Dewar it would appear that the University intends to maintain an attitude of opposition. Confirmation of this view is also found in the fact that the new Veterinary College in the same city has become affiliated to the University of Liverpool. The demands which the Government and the local health authorities now make on members of the veterinary profession are considerable and to meet these a higher standard of attainment is certainly necessary. It seems therefore only right that, granted the fulfilment of appropriate conditions, veterinary students should have the opportunity of obtaining the hall-mark of a University degree.

